# Role of Virtual Bronchoscopy in Evaluation of Suspected Foreign Body in Children's Tracheobronchial Tree

**DOI:** 10.1055/s-0043-1778015

**Published:** 2024-02-05

**Authors:** Rachana M. Prajapati, Jayman B. Raval, Ranjan G. Aiyer

**Affiliations:** 1 Department of Ear, Nose & Throat and Head and Neck Surgery, GMERS Medical College, Gotri, Vadodara, Gujarat, India; 2 Department of Ear, Nose & Throat and Head and Neck Surgery, Baroda Medical College, Vadodara, Gujarat, India

**Keywords:** bronchoscopy, foreign bodies, aspiration

## Abstract

**Introduction** The presence of foreign bodies in the airways remain a diagnostic challenge to healthcare professionals. They can become life threatening emergencies that require immediate intervention or go unnoticed for weeks and even months. Prevention is best but early recognition remains a critical factor in treatment of foreign body inhalation in children.

**Objective** To study the diagnostic advantages of virtual over rigid bronchoscopy in the evaluation of children with suspected foreign body in the tracheobronchial tree and plan for early management.

**Methods** A crossectional study conducted at a tertiary care hospital & medical college in India. A total 24 patients (0-12-years-old) who presented with complaints of sudden onset of coughing, choking, and breathing difficulty were included during the 2-year duration, from January 2018 to December 2019. All patients underwent virtual and rigid bronchoscopy.

**Results** In 8 patients, foreign bodies detected by virtual bronchoscopy were confirmed by rigid bronchoscopy. There was one case in which virtual bronchoscopy showed no foreign body, but rigid bronchoscopy detected it. In 15 cases virtual and rigid bronchoscopy did not show foreign bodies. The sensitivity, specificity, positive and negative predictive value of virtual bronchoscopy were 88.88, 100, 100, and 93.75%, respectively.

**Conclusions** Virtual bronchoscopy is less invasive and does not require general anesthesia but cost and availability are limitations. It can be used as method of investigation in children with suspected foreign body aspiration.

## Introduction

Chevalier Jackson 1 defined a foreign body as “an object or a substance that is foreign to its location”. According to Fidkowski et al., foreign body aspiration is one of the most common causes of accidental death in children under the age of 4. 2

In suspicious of foreign body aspiration, neck and thorax radiographs are essential. In the first 24 hours of aspiration, most of the time radiographs are normal but can be abnormal as duration passes. 1 The presence of a foreign body in the airway can lead to obstruction and/or complications like lung infection, atelectasis, destruction of the lung parenchyma, bronchiectasis, and even death. Timely diagnosis and removal is necessary for prevention of complications. 3 4 5 6 If there is suspicion of foreign body aspiration, bronchoscopy is advised for definitive diagnosis and treatment.

Computed tomography (CT) virtual bronchoscopy is software based; three-dimensional (3D) visualization formats created from noninvasive medical imaging methods have the goal of creating views similar to a minimally invasive bronchoscopy procedure. It offers a detailed image of the tracheobronchial tree, with minimal risk of infection or perforation, which helps preoperative planning for airway interventions that would otherwise not be possible. 7 When chest radiography is not conclusive and there is a positive clinical diagnosis, CT virtual bronchoscopy must be considered.

Our objective was to study the advantages of virtual over rigid bronchoscopy in the evaluation of children with suspected foreign body in the tracheobronchial airway, and to plan for timely management.

## Methods

This crossectional study was carried out in an Indian tertiary level hospital & medical college, between January 2018 to December 2019. There was a total of 24 patients, from 0 to 12-years-old who presented in the pediatric and ear, nose & throat (ENT) outpatient department, emergency, and ward with complaints of sudden onset of coughing, choking, and breathing difficulty were included in the study. Patients older than 13 years, foreign body already confirmed on chest radiography, presenting with cerebral hypoxia secondary to foreign body aspiration, and history of foreign body in patients with severe respiratory distress were excluded.

A detailed history regarding complaints of sudden onset of coughing, choking while playing, difficulty in breathing, fever, stridor, current illness, and family history were taken into consideration. History of any obvious foreign body inhalation was asked from parents and elder siblings. A detailed clinical examination of the nose and throat was done, as well as general physical, systemic, and detailed respiratory system examinations for air entry and abnormal sounds. Oxygen saturation (SpO2) was checked. All patients underwent chest radiographs. Preoperative anti-inflammatory and injectable steroids were given to reduce airway inflammation and oedema. All patients underwent rigid bronchoscopy under general anesthesia.

A CT virtual bronchoscopy was performed in all cases, through a helical CT scanner with 2-mm collimation, 1.5 pitch, 120 kV, 100 mA, and a gantry rotation speed of 360°/sec. All images were reconstructed at 1mm intervals using a standard reconstruction algorithm into 3D virtual images. The image generation was performed in three stages. By using the workstation computer, navigation through the tracheobronchial tree was performed interactively. The axial images, as well as virtual bronchoscopic ones, were assessed simultaneously and then reported by the radiologist.

After adequate preoperative measures, a rigid bronchoscopy was performed in the operation room with use of general anesthesia and a rigid pediatric bronchoscope (Karl Storz, Tutligen, Germany), calibers 3, 4, 5, & 6.5 according to the patients' age and built.. The whole respiratory tract was carefully examined for the presence of foreign bodies, inflammation, anatomic abnormalities, purulent secretions, and granulation tissue. The removal was done by using an alligator jaw and a cup forceps. The results of rigid bronchoscopy were then compared with virtual bronchoscopy images.

## Results

In this study, a total 24 patients were included. Among them, 17 patients (70.84%) were 0 to 3-years-old, 4 (16.67%) were between 3 to 6-years-old, 3 patients (12.5%) were in the 6 to 9-years-old age group. The most common age group was the 0 to 3-years-old. Out of 24 cases, 20 (83.33%) were male and 4 (16.67%) were female, which showed a male preponderance, the M:F ratio being 5:1. The most common presenting symptoms were cough (n = 14, 54.83%) and breathing difficulty (n = 12, 50%). While 10 patients (41.66%) had fever as presenting symptom. On chest radiograph examination, 10 patients (41.67%) had consolidation, 3 (12.5%) had lung collapse, and 1 had pleural effusion. For 11 patients (45.84%) had no abnormalities on the chest radiograph.

**Fig. 1:**
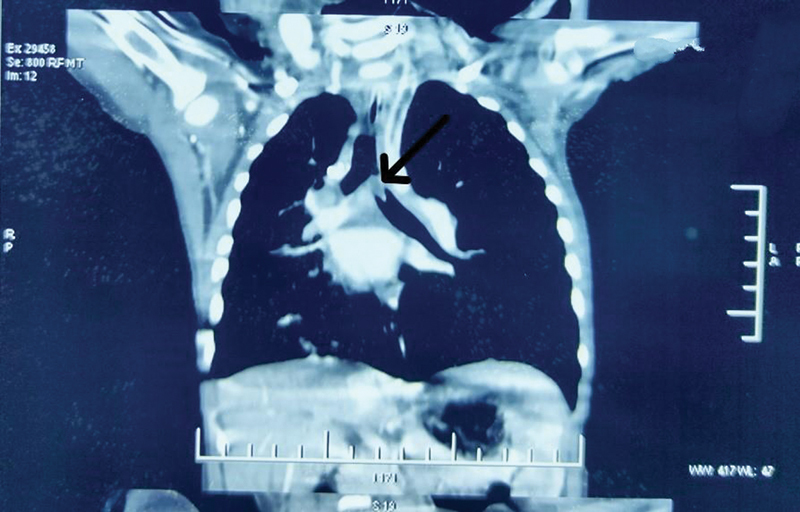
Foreign body in left main bronchus on a coronal CT image (arrow).

**Fig. 2:**
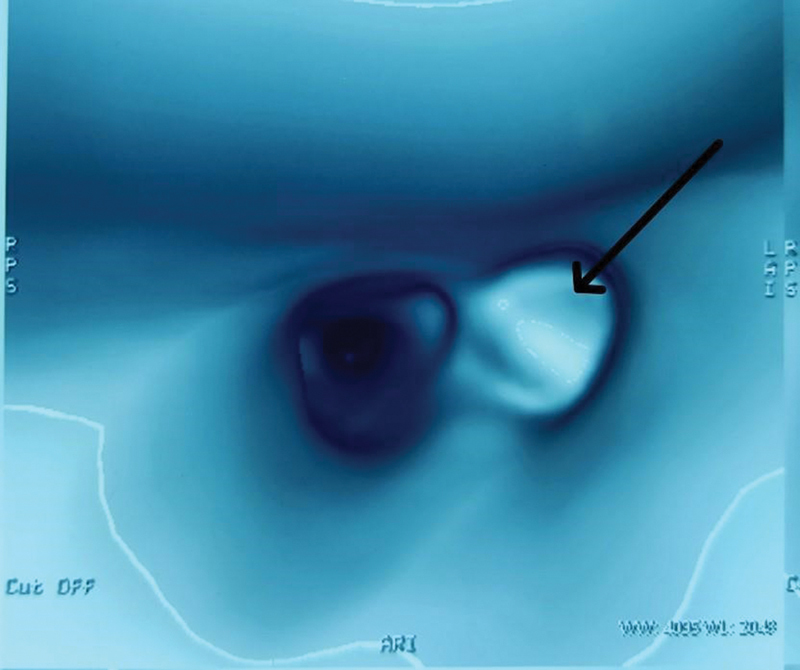
Virtual bronchoscopy image of foreign body located in the left main bronchus (arrow).

**Fig. 3:**
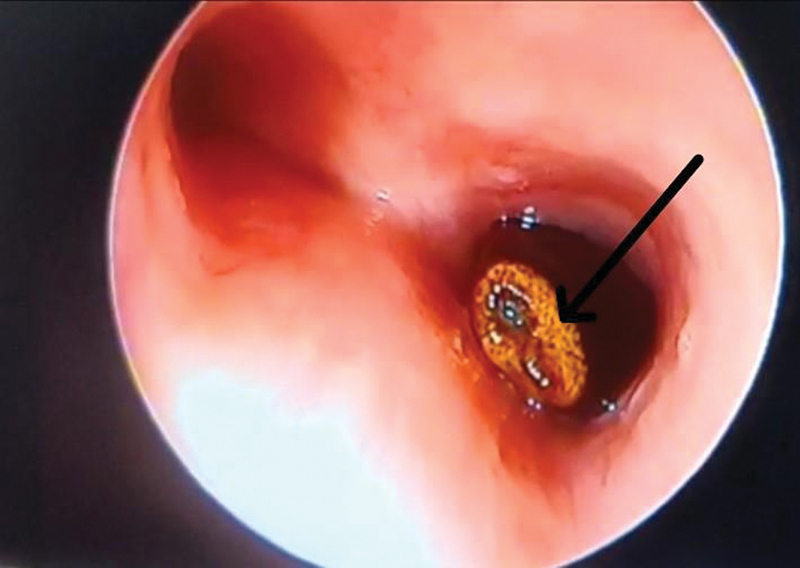
Foreign body seen in left main bronchus during rigid bronchoscopy (arrow).

In virtual bronchoscopy, a foreign body was detected in 8 patients (33.34%), while 16 (66.66%) of them had none. Figure 1 shows a coronal CT image in which the foreign body is in left main bronchus. Figure 2 shows an image of a virtual bronchoscopy in which the foreign body is in the left main bronchus. Out of 24 cases, 18 had other changes like consolidation or collapse of the lung lobe, ground glass density in the lungs, retained secretions in the bronchus, and pleural effusion. Furthermore, 9 cases (37.50%) had foreign body in the rigid bronchoscopy and 15 cases (62.50%) had none. Figure 3 shows a foreign body in left main bronchus during rigid bronchoscopy in the same case as mentioned above. There were also 10 cases with other changes like excessive mucus in bronchus, mucus plug in the bronchus, slough in the subglottic region, subglottic membrane, subglottic narrowing, and polypoidal lesion the in lateral wall of trachea. The removed foreign bodies were a custard apple seed, a ground nut, a tamarind seed, a peanut, and a sugarcane husk.

Virtual and rigid bronchoscopy were positive (true positive) for foreign body in 8 patients, and both were negative (true negative) in 15. In only 1 case, virtual bronchoscopy was negative and rigid bronchoscopy was positive for foreign body (false negative). There were false positive cases in which virtual bronchoscopy was positive and rigid bronchoscopy was negative for foreign body ( Table 1 ).

**Table 1:** Comparison of results between virtual bronchoscopy and rigid bronchoscopy.

		Rigid bronchoscopy (foreign body removed)		Total
		Positive	Negative	
**Virtual bronchoscopy (Foreign body detected)**	Positive	8 (True positive)	0 (False positive)	8
	Negative	1 (False negative)	15 (True negative)	16
**Total**		9	15	24

We calculated the sensitivity (88.88%), specificity (100%), positive (100%), and negative predictive value (93.75%) of virtual bronchoscopy . To measure intertest variability, the Cohen kappa test was used the results obtained were: kappa = 0.909, standard error = 0.0886, and 95% confidence interval = 0.735 to 1.

The kappa value measures the proportion of nonrandom agreement between two tests or two observers. As a rule of thumb, results greater than 0.75 are considered excellent and lower than 0.40 are poor. Since the kappa value obtained in the test above is 0.909, there is a strong or excellent agreement between the tests used in this study, so virtual bronchoscopy can be used as a method of investigation in children with suspected foreign body aspiration.

## Discussion

In day-to -ay otorhinolaryngological practice, aspiration of foreign body into the tracheobronchial tree is a common problem, seen especially in children. The swallowing reflex is much less developed in children and during inspiration objects are more easily inhaled. Furthermore, this is often misdiagnosed and treated as respiratory tract infection/pneumonia, asthma, or laryngitis, which can lead to complications.

Most commonly, the incidence of foreign body aspiration (70.84%) was seen in children from 0 to 3-years-old in this study. Our findings match those of François et al., who had 71% children younger than 3-years-old. 8 The study by Srivastava et al. had 61% children between 1 and 5 years of age, 9 and the male to female ratio was 4:1, which was similar to our study. 9 It is understood that male children are more playful, active and mischievous. The majority of patients in our study are from lower socioeconomic status. Lack of social education, poor personal habits, and parental awareness are also important factors.

Our study's findings are similar to those of Kugelman et al., who also mentioned that the most common presenting symptoms of foreign body aspiration are choking and acute cough. 10 Burton et al. identified cough and wheezing as the most common symptoms. 11 Shubha et al. observed that the clinical triad of cough, respiratory distress, and stridor was highly predictive of foreign body aspiration. 12 In our study, the most common symptoms were also coughing (54.83%) and breathing difficulty (50%).

We observed that chest X-ray was normal in as many as 45.84% of the cases. Therefore, foreign body in airway can't be ruled out by a negative chest X-ray. In our study, we included suspected cases of foreign bodies in which chest radiograph did not identify them, so those cases were evaluated further by 3D CT scan with virtual bronchoscopy. All cases underwent rigid bronchoscopy, and foreign bodies were removed in 9 of them. In the study of Williams et al., the nature of removed foreign bodies was organic materials, including nuts and seeds, which was similar to our study. 13 Ramírez-Figueroa et al. also showed that 64.4% foreign bodies were organic in nature—mainly peanut, seeds and beans—while 35.6% were inorganic. 14 There was one case in which no foreign body was detected on virtual bronchoscopy but, when the patient underwent rigid bronchoscopy, a sugarcane husk was found in the left main bronchus with associated lung changes. Long standing neglected foreign bodies with secondary lung changes, such as diffuse consolidation, cavities in the left lung, and pleural effusion, might be the cause for missed foreign body on virtual bronchoscopy.

The sensitivity and specificity of virtual bronchoscopy compared with rigid bronchoscopy were 88.88 and 100%, respectively. In the study by Haliloglu et al., the sensitivity and specificity of CT virtual bronchoscopy, compared with rigid bronchoscopy, were 100%, 15 similar to our study. According to various studies, the sensitivity of multidetector computed tomography (MDCT) scan for the detection of bronchial foreign bodies is close to 100%, with a specificity between 66.7 and 100%. 16 Virtual bronchoscopy had a 100% positive and 93.75% negative predictive value, in our study. Hassan et al. showed that MDCT with a positive predictive value of 75% and negative of 100%. 17

Although virtual bronchoscopy is not used for removal of foreign body directly, it can provide an accurate location of the foreign body, which is helpful in the preparation prior to surgical removal through rigid bronchoscopy. A negative virtual bronchoscopy can prevent the patient from undergoing surgery and unnecessary hazards of general anesthesia, since it is the only imaging modality with 99.9% reassurance about the presence or absence of foreign body. 18 When clinical diagnosis strongly suspects aspiration of foreign body and chest radiograph is normal, virtual bronchoscopy can be considered a diagnostic, life-saving tool.

## Conclusion

Virtual bronchoscopy is a non-invasive modality of investigation for pediatric patients with suspicions of foreign body aspiration that does not require general anesthesia. However, the cost of virtual bronchoscopy and unavailability of its software in peripheral centers are limitations. As for rigid bronchoscopy, it is the gold standard diagnostic and treatment method for tracheobronchial foreign body. Virtual bronchoscopy provides a proper non-invasive image and location of the foreign body in the tracheobronchial tree preoperatively, thus reducing the duration of removal by rigid bronchoscopy.
